# Comprehensive transcriptomic meta-analysis unveils new responsive genes to methyl jasmonate and ethylene in *Catharanthusroseus*

**DOI:** 10.1016/j.heliyon.2024.e27132

**Published:** 2024-02-26

**Authors:** Seyede Nasim Tabatabaeipour, Behrouz Shiran, Rudabeh Ravash, Ali Niazi, Esmaeil Ebrahimie

**Affiliations:** a Department of Plant Breeding and Biotechnology, Faculty of Agriculture, Shahrekord University, Shahrekord, Iran; b Institute of Biotechnology, Shahrekord University, P.O. Box 115, Shahrekord, Iran; c Department of Biotechnology, Faculty of Agriculture, Shiraz University, Shiraz, Iran; d School of Animal and Veterinary Sciences, The University of Adelaide, Adelaide, SA 5371, Australia

**Keywords:** *Catharanthus roseus*, RNA-Seq data, Meta-analysis, Co-expression analysis, Hormones, Metabolites

## Abstract

In *Catharanthus roseus*, vital plant hormones, namely methyl jasmonate (MeJA) and ethylene, serve as abiotic triggers, playing a crucial role in stimulating the production of specific secondary compounds with anticancer properties. Understanding how plants react to various stresses, stimuli, and the pathways involved in biosynthesis holds significant promise. The application of stressors like ethylene and MeJA induces the plant's defense mechanisms, leading to increased secondary metabolite production. To delve into the essential transcriptomic processes linked to hormonal responses, this study employed an integrated approach combining RNA-Seq data meta-analysis and system biology methodologies. Furthermore, the validity of the meta-analysis findings was confirmed using RT-qPCR. Within the meta-analysis, 903 genes exhibited differential expression (DEGs) when comparing normal conditions to those of the treatment. Subsequent analysis, encompassing gene ontology, KEGG, TF, and motifs, revealed that these DEGs were actively engaged in multiple biological processes, particularly in responding to various stresses and stimuli. Additionally, these genes were notably enriched in diverse biosynthetic pathways, including those related to TIAs, housing valuable medicinal compounds found in this plant. Furthermore, by conducting co-expression network analysis, we identified hub genes within modules associated with stress response and the production of TIAs. Most genes linked to the biosynthesis pathway of TIAs clustered within three specific modules. Noteworthy hub genes, including Helicase ATP-binding domain, *hbdA*, and *ALP1* genes within the blue, turquoise, and green module networks, are presumed to play a role in the TIAs pathway. These identified candidate genes hold potential for forthcoming genetic and metabolic engineering initiatives aimed at augmenting the production of secondary metabolites and medicinal compounds within *C. roseus.*

## 1 Introduction

Plants represent a promising reservoir of medicinal compounds, utilized for therapeutic objectives. These compounds, known as secondary metabolites, are naturally synthesized by plants as a defense mechanism against stress factors [1]. Various environmental factors, abiotic stresses, and the application of different exogenous chemicals as elicitors influence the quantities of diverse secondary metabolites in plants. Methyl jasmonate (MeJA) and ethylene, two plant hormones functioning as abiotic triggers, play a crucial role in signaling the production of specific secondary metabolites by regulating various target genes and biochemical compounds [2,3]. Secondary metabolites are commonly grouped into three categories: alkaloids, phenols, and terpenoids [4]. Among these, alkaloids represent a significant class, comprising diverse nitrogen-based compounds with relatively low molecular weights [5]. Terpenoid indole alkaloids (TIA), a prominent subset within the alkaloid family, possess valuable therapeutic properties, particularly in the treatment of diseases like cancer, making them highly relevant on a global scale. TIAs, predominantly found in three plant families “Apocynaceae, Rubiaceae, and Loganiaceae” have been extensively researched [6]. Within the Apocynaceae family, *Catharanthus roseus* stands out as a significant medicinal plant, harboring approximately 130 TIAs. Among these compounds, vinblastine and vincristine, considered highly potent anti-cancer agents, are widely utilized in various cancer treatments involving chemotherapy [7]. However, these crucial chemicals are exclusively derived from *C. roseus*, and their limited and restrained accumulation in the plant fails to meet the global demand [5].

**Fig. 1:**
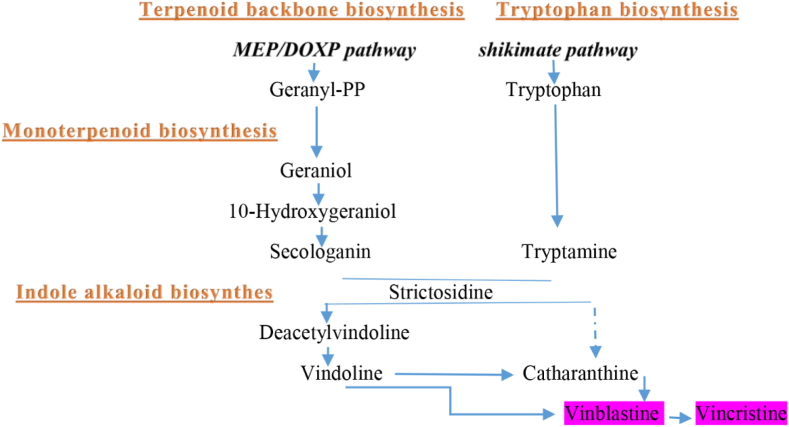
Diagram depicting the biosynthetic pathways of terpenoid indole alkaloids (TIAs) in *C. roseus* (Adapted from Ref. [4]).

In recent times, efforts have been made in utilizing genetic and metabolic engineering techniques to boost the production of TIAs. However, advancements in this area have been hindered by the intricate pathways associated with TIAs (as depicted in Fig. 1) and the limited discovery of key genes linked to their accumulation. Consequently, there's an urgent need for research aimed at uncovering new genes responsible for both the generation and control of TIAs [4]. Analyzing the pattern of gene expression stands out as a crucial method in identifying promising candidate genes for this purpose. Advancements in understanding new genes and assessing gene expression in secondary metabolite production have been facilitated by RNA-seq technology. Successful utilization of this method has been evident in research focused on medicinal plants [8,9]. Nevertheless, this approach is not without limitations. Factors such as the expensive nature of RNA-seq, coupled with constraints in conducting a limited number of experiments and repetitions per research project, hinder comprehensive data collection due to imposed restrictions [10].

Meta-analysis stands out as a potent approach in uncovering new DEGs by enhancing sample sizes and statistical robustness. This method combines transcriptome data across multiple experiments, pinpointing the primary gene set governing various traits. Consequently, it becomes feasible to strength existing data to identify crucial elements within biological processes, notably those linked to secondary metabolism. By doing so, this technique boosts the reliability of transcriptomic data and mitigates the impact of environmental fluctuations attributed to the limited number of biological and experimental repetitions in individual studies [11,12]. In this study, we conducted a meta-analysis of RNA-seq data in *C. roseus*, focusing on two plant hormones -MeJA and ethylene-as abiotic elicitors. Our aim was to pinpoint novel DEGs by employing functional enrichment analysis across pathways, transcription factors, protein kinases, and microRNA families. Furthermore, we utilized a systems biology approach to identify essential genes acting as hubs and gene networks that potentially influence the TIA biosynthetic pathways responsible for generating secondary metabolites, notably anticancer drugs. To our knowledge, our research marks the initial exploration involving RNA-Seq meta-analysis across multiple datasets stimulated by various hormones in *C. roseus*.

## 2 Materials and methods

### 2.1 RNA-seq data collection

In the winter of 2021, a search was conducted within the SRA repository of the NCBI database to identify RNA-Seq studies focusing on *C. roseus* responses to MeJa and ethylene hormones. This search employed specific criteria, including the requirement for datasets to be RNA-seq gene expression profiles generated via the Illumina HiSeq platform. Moreover, the inclusion criteria encompassed samples featuring both control and hormone-treated groups, with a paired library layout. Subsequently, three studies meeting these criteria were identified through key indicators. The Fastq files of selected samples meeting these criteria were sourced from the ENA Nucleotide database, accessible as paired-end Illumina reads. The studies can be accessed under study accessions PRJNA185483 and PRJNA358259 (since this study involved two distinct hormonal stimuli, each was considered as an individual study), each associated with distinct treatments (refer to Table S3 for details). The Fastq files of these chosen samples were obtained from the ENA Nucleotide database and were publicly available.

### 2.2 Preprocessing of RNA-seq data

Before mapping the reads, preliminary processing was conducted on the RNA-Seq data. To ensure the dataset's reliability, an initial integrity check was performed. FastQC, a tool by Andrews (2010), was utilized for an in-depth quality assessment, focusing particularly on per-base sequence quality and adapter content [13]. Subsequently, Trimmomatic, employing default parameters as outlined by Bolger et al. (2014), was employed to cleanse the fastq files [14].

### 2.3 Reads mapping and expression quantification

Transcript accumulation quantification involved a series of three procedures, all executed via STAR: initially generating the genome index, followed by mapping the reads to this index as a reference genome, and ultimately totaling the read count per gene. Reads were mapped to the generated genome reference, which integrated both the genome assembly (ASM94934v1) [15] and the annotation in GFF3 format of *C. roseus*, retrieved from Dryad (https://doi.org/10.5061/dryad.08vv50n) [16]. The counting process was conducted using STAR (v 2.7.0) with default settings [17]. Throughout these analyses, the Linux terminal, specifically Ubuntu 20.04, served as the operating platform. The resultant gene counts were subsequently employed in subsequent stages of analysis.

### 2.4 Batch effect correction

ComBat-seq was employed to address batch effects correction, specifically chosen due to its suitability for handling RNA-seq read count data where both the input and output manifest as integer counts [18]. Accordingly, we utilized the raw count data matrix, devoid of any prior normalization, as the input for batch effect correction via ComBat-seq, implementing default parameters for this process.

### 2.5 Gene functional annotation

All gene sequences underwent annotation utilizing various databases, including Nr (NCBI non-redundant protein sequences), KOG/COG (Clusters of Orthologous Groups of Proteins), Swiss-Prot protein, InterPro, and TAIR. This annotation process utilized BLASTX with a specified E-value of ≤10−5. To further enhance their annotation, we employed the Blast2GO program v 6.0.3 [19]. This step involved assigning GO terms, an EC number, and KEGG information based on the BLAST results.

### 2.6 Identification of genes involved in the biosynthesis of TIAs

Various blast methods and databases were employed to identify CRO-genes associated with the TIAs pathways in *C. roseus*, which are responsible for producing vinblastine and vincristine. These pathways include terpenoid backbone biosynthesis [PATHWAY: ko00900], monoterpenoid biosynthesis [PATHWAY: ko00902], phenylalanine, tyrosine, and tryptophan biosynthesis [PATHWAY: ko00400], and indole alkaloid biosynthesis [PATHWAY: ko00901], all fundamental to TIAs biosynthesis. Through comprehensive database searches and annotations utilizing the KEGG databases, all unigenes were analyzed and annotated for genes linked to the synthesis of TIAs.

### 2.7 Differential expression

Once genes with low counts were filtered out, the read counts underwent integration into edgeR (v3.16.5) [20]. Normalization across all samples was carried out utilizing the calcNormFactors function. Differential expression analysis was conducted between treatment and control read counts within each RNA-Seq study. Genes exhibiting significant differential expression were identified using criteria of P-value <0.05 and log2FC ≤ −1 or log2FC ≥ 1.

### 2.8 Meta-analysis

This study involved conducting a meta-analysis using RNA-Seq data obtained from MeJa/ethylene studies. Table S3 provides an overview of the essential characteristics of each study and dataset. A total of three studies were examined in this meta-analysis, encompassing 10 treatment and 7 control samples. To perform the meta-analysis, the metaRNASeq package was utilized [11]. In metaRNASeq, Fisher's combined probability test is employed for meta-analyses, a method widely used in diverse studies. This approach combines p-values for each gene across multiple studies using the formula: F_g_ = −2 $\sum_{s=1}^{s}\ln$ (p_gs_), where pgs denotes the raw p-value for a gene (g) in a specific study (s). F_g_ follows a chi-squared distribution with 2S degrees of freedom due to the independent p-values. Smaller p-values correspond to larger F_g_ values, leading to the rejection of the null hypothesis. To address multiple testing issues, the Benjamini-Hochberg false discovery rate (FDR) was applied for p-value adjustments, considering an adjusted p-value of 0.05 as statistically significant. In this meta-analysis, differentially expressed genes (DEGs) were identified as genes with an FDR of 0.05 and an average fold change (FC) of ≥2 (log2FC ≤ −1 or log2FC ≥ 1). The resulting DEGs were then utilized for subsequent analyses.

### 2.9 Annotation of DEGs using Gene ontology and pathway analysis

The gene ontology (GO) analysis for differentially expressed genes (DEGs) was conducted using the Blast2GO program v 6.0.3 [19] and the DAVID web server [21]. This analysis aimed to categorize GO functions based on biological process, molecular function, and cellular component, with a significance threshold set at p-values <0.05. Furthermore, the Blast2GO tool, in combination with the Kyoto Encyclopedia of Genes and Genomes (KEGG) database, was utilized to identify key pathways.

### 2.10 Transcription factor (TF), protein kinase (PK), and microRNA families identification

The iTAK database (http://bioinfo.bti.cornell.edu/cgi-bin/itak/index.cgi) was utilized for the identification and categorization of transcription factors (TFs) and protein kinases (PKs) [22]. Furthermore, cytoscape version 3.9.1 was employed to construct interaction networks between DEGs-TFs/DEGs-PKs [23]. The top 5 TFs and PKs identified were regarded as the primary transcriptional regulators of the DEGs.

The miRNA sequences were employed as queries for the MepmiRDB database (http://mepmirdb.cn/mepmirdb/index.html) and psRNATarget server (http://plantgrn.noble.org/psRNATarget/) using default settings, except for adjusting the maximum expectancy to 2. This modification aimed to identify potential miRNAs associated with the DEGs. Given the incomplete identification and relatively limited understanding of *C. roseus* miRNAs and their functions, the sequences obtained from MepmiRDB were aligned against Arabidopsis thaliana miRNAs.

### 2.11 *Cis*-elements analysis

Using Ensembl Plants (http://plants.ensembl.org), we extracted the 1 kb upstream flanking regions of the differentially expressed genes (DEGs). These sequences underwent analysis in MEME (meme.nbcr.net/meme/intro.html) using default settings, with a few adjustments: specifically, allowing a maximum of 10 motifs and setting a threshold E-value of <1e-4 [24]. To identify conserved motifs, we referenced the JASPAR CORE 2018 plants motif database [25] and employed the Tomtom v 5.0.1 tool (http://meme-suite.org/tools/tomtom) [26] with an E-value cut-off of 0.05 to define known *cis*-regulatory elements (CREs). Additionally, for inferring potential motif functions, we utilized the GoMo tool (http://meme-suite.org/tools/gomo) [27].

### 2.12 Analysis of Gene Co-expression networks

In search of networks exhibiting related expression patterns among DEGs, we conducted a weighted gene co-expression network analysis (WGCNA) [28] using the normalized expression values matrix encompassing all samples. This involved constructing a similarity matrix by calculating Pearson correlations between every pair of DEGs. To transform this into an adjacency matrix, we utilized a power function. Additionally, a topological overlap matrix (TOM) was generated for hierarchical clustering analysis. Finally, we employed a dynamic tree-cut method to identify modules displaying co-expression among genes. Modules were established with a cut height set at 0.975 and a minimum module size of 30 genes. Subsequently, connectivity scores were computed, identifying genes with the most significant connections within each module, designated as hub genes. Using the CytoHubba tool within Cytoscape software v3.9.1, the hub genes within the chosen modules were identified. Further computational analysis of node connections utilized the Maximal Clique Centrality (MCC) tool. Additionally, the DAVID web server was employed to conduct GO functional analysis on the main modules.

### 2.13 Plant materials and hormonal treatments

Seeds of *C. roseus* from the PanAmerican Seed Company (Lot 2021599001, A21-082-G1) were acquired via Bazrco in Iran. Before planting, the seeds underwent sterilization using a solution of 70% ethanol and 1% sodium hypochlorite, followed by rinsing with distilled water. These seeds were germinated in seedling trays filled with perlite and later transferred to pots containing a mixture of sand, soil, and leaf mold in a ratio of 1:1:2. Cultivation took place in a greenhouse with three replicates. Initially, seedlings were nourished using a modified Hoagland's solution until they reached the 6-8-leaf stages. Subsequently, hormonal treatments involving ethylene (E), methyl jasmonate (M), and a combination of both (EM) were applied to the seedlings. Four time points—0 (control), 8, 24 h, and 1 week—were considered for each treatment. Methyl jasmonate treatment utilized a concentration of 150 μM, while ethephon at 100 μM was used for ethylene treatment via leaf spraying. Following treatments, leaf material from both treated and control plants were sampled and promptly frozen in liquid nitrogen and stored at −80 °C.

### 2.14 Validation by RT-qPCR

Four genes *CRO_T120028* (*DAT*), *CRO_T131457* (*TSB*), *CRO_T138994* (*CYP76A26*), and *CRO_T107712* (*AACT*) related to the biosynthetic pathway of TIAs at different points of the pathway to the production of vincristine and vinblastine, which were identified as DEGs through meta-analysis, were selected. The first three genes showed an upregulation and the fourth gene downregulated, respectively. They were chosen to validate their expression based on the meta-analysis findings. Total RNA from leaf tissue was extracted using RNX-Plus Solution (SinaClon Co., Tehran, Iran) following the manufacturer's guidelines. DNase treatment with DNaseI was subsequently carried out. The YTA Reverse Transcriptase Kit was employed to synthesize cDNA, following the manufacturer's instructions. Real-time quantitative PCR (RT-qPCR) was conducted using the YTA SYBR Green PCR Master Mix whit two biological and technical replications. For normalization of relative expression levels, the RPS9 gene served as a reference. Primer sequences for RT-qPCR are available in Table S12.

## 3 Results

### 3.1 Functional annotation of all unigenes

**Fig. 2:**
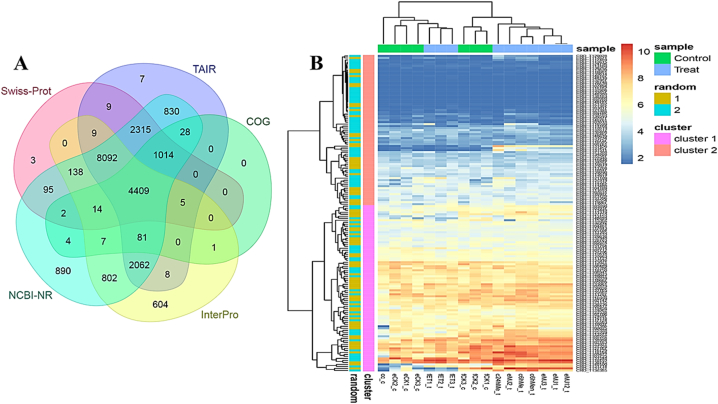
(A) Venn diagram illustrating the overlap of annotated unigenes from five distinct databases using an E-value threshold of ≤10^−5^ and (B) heatmap representing the expression pattern of CRO-Genes associated with the TIA pathway.

Out of 34,363 unigenes, 22,814 were found to be expressed in the samples, while the remainder were not detected. To annotate these unigenes, a BLAST search (E-value ≤10-5) was conducted against several public databases, including Nr, Swiss-Prot, TAIR, InterPro, and COG (refer to Table S1). Among the total unigenes, 4409 (19.32%) showed significant matches across all databases (depicted in Fig. 2A). Additionally, all unigenes underwent annotation related to TIAs biosynthesis by referencing the KEGG databases (Table S2). Subsequently, BLAST against various databases identified 159 CRO-genes associated with the TIA pathway, showcased in a heatmap depicted in Fig. 2B.

### 3.2 Utilizing meta-analysis for identifying DEGs

**Fig. 3:**
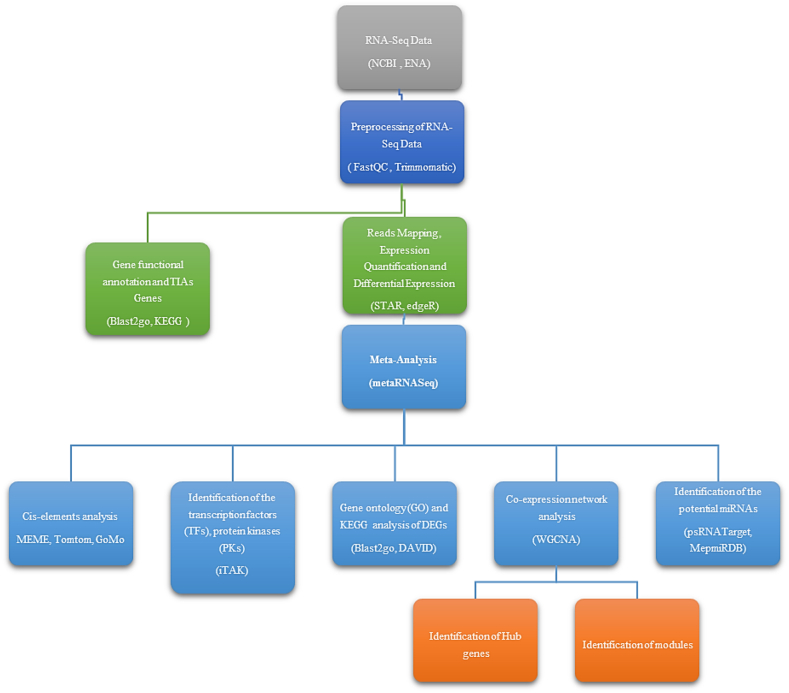
The workflow of the meta-analysis and downstream stages in the investigation of *C. roseus* under hormone treatments.

Using meta-analysis, we investigated the *C. roseus* gene expression responses to ethylene (ET) and methyl jasmonate (MeJA), as outlined in Table S3. Fig. 3 illustrates a schematic detailing the key stages of this study.

**Fig. 4:**
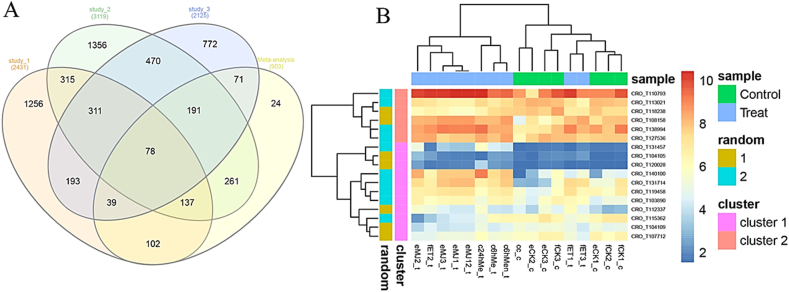
(A) Venn diagram illustrating the count of differentially expressed genes (DEGs) derived from individual studies versus the meta-analysis, and (B) heatmap demonstrating the expression pattern of DEGs found in the TIA pathways as identified through the meta-analysis.

The meta-analysis incorporated three research investigations comprising a total of seventeen samples. In each study, the samples were categorized into control and treatment groups to investigate variations in gene expression. Following data preprocessing, corrections for batch effects were applied to the gene expression data to reduce inconsistencies across studies. The correction of batch effects notably minimized differences in batches between datasets, as observed from the PCA and MA plot outcomes (Fig. S1). Through the meta-analysis, 903 genes were identified as significantly differing between conditions, with 24 of these genes being identified as differentially expressed genes (DEGs) not previously detected in any of the individual studies (Fig. 4A). In the datasets, 534 genes showed significant increases in expression, whereas 369 genes displayed decreased expression levels (Table S4). Among the identified differentially expressed genes (DEGs), notable upregulations were observed in genes related to Germin-like protein subfamily T member 1 (*GLP1*), Monoterpene synthase 7 (*TPS7*), Alpha-terpineol synthase (*ATESY_VITVI*), Pathogenesis-related genes transcriptional activator (*PTI5*), Secoisolariciresinol dehydrogenase (*SIRD*), *CRO_T122623*, and *CRO_T131457*. Conversely, the most prominent downregulations were seen in Xyloglucan endotransglucosylase/hydrolase protein 9 (*XTH9*), Expansin-A1 (*EXPA1*), *CRO_T110204*, Protein DMR6-LIKE OXYGENASE 2 (*DLO2*), and *CRO_T104942* (Table S4). Furthermore, within the DEGs associated with TIAs biosynthetic pathways, 17 genes displayed distinct expression patterns, as depicted in the heatmap (Fig. 4B). Among these genes involved in TIAs biosynthesis, 12 exhibited increased expression while 5 showed decreased expression. Specifically, *CRO_T131457*, *CRO_T138994*, and *CRO_T120028* were among the upregulated genes, whereas CRO_T107712 was identified as a downregulated gene, selected for confirming the expression patterns from the meta-analysis results. Fig. S3 illustrates the biosynthetic pathways associated with the production of TIAs for each gene, highlighting the enriched enzymes coded by these genes.

### 3.3 DEGs' gene ontology (GO) and KEGG pathways analysis

**Fig. 5:**
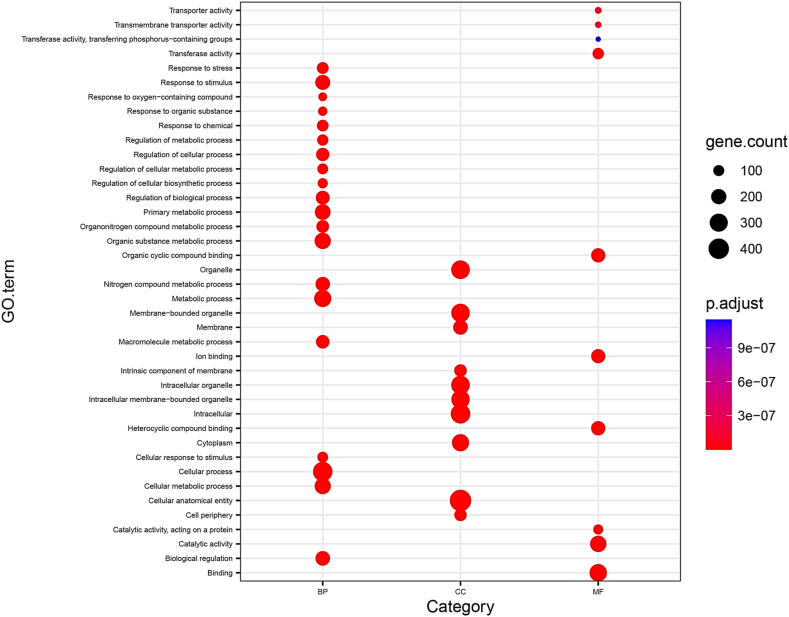
The Gene Ontology (GO) analysis associated with the differentially expressed genes (DEGs) in *C. roseus* subjected to hormone treatments.

To unravel and categorize the roles of the differentially expressed genes (DEGs), we employed Blast2GO and the DAVID web server to assign Gene Ontology (GO) terms to the entire set of DEGs. In the general category of biological processes, terms such as ‘Cellular process (GO:0009987)’, ‘Metabolic process (GO:0008152)’, ‘Cellular metabolic process (GO:0044237)’, ‘Organic substance metabolic process (GO:0071704)’, and ‘Response to stimulus (GO:0050896)’ emerged as the most significantly enriched GO terms. Within the cellular component category, prevalent GO terms among the DEGs included ‘Cellular anatomical entity (GO:0110165)’, ‘Intracellular (GO:0005622)’, and ‘Organelle (GO:0043226)’. As for molecular functions, the most prevalent terms were ‘Ion binding (GO:0043167)’, ‘Catalytic activity (GO:0003824)’, and ‘Binding (GO:0005488)’ (Fig. 5).

**Fig. 6:**
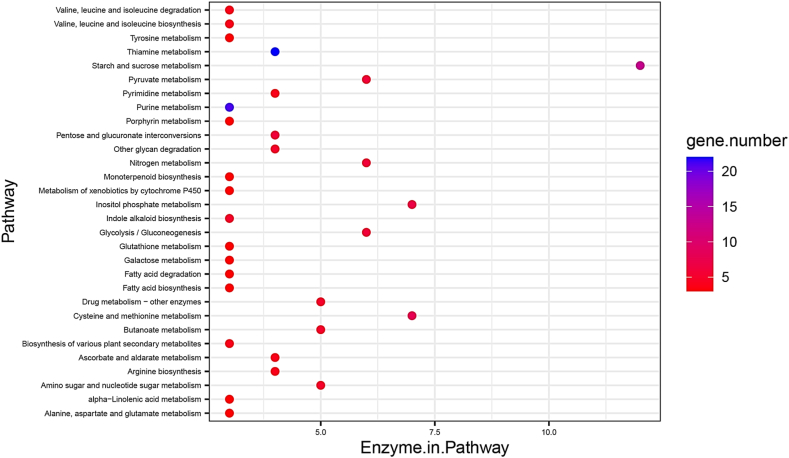
The KEGG pathways associated with the differentially expressed genes (DEGs) in *C. roseus* in response to hormone stimuli.

The analysis of differentially expressed genes' KEGG pathways revealed significant enrichment, with the top five pathways identified by Blast2GO based on the number of enzymes within each pathway: "Starch and sucrose metabolism KO00500 (12 enzymes, 13 genes)," "Cysteine and methionine metabolism KO00270 (7 enzymes, 8 genes)," "Inositol phosphate metabolism KO00562 (7 enzymes, 7 genes)," "Glycolysis/Gluconeogenesis KO00010 (6 enzymes, 6 genes)," and "Pyruvate metabolism KO00620 (6 enzymes, 6 genes)" (Fig. 6).

From the DEGs identified through the meta-analysis, employing KEGG pathway analysis via Blast2GO, we identified 11 genes associated with the biosynthetic pathways of TIAs. These genes contribute to various pathways such as Terpenoid backbone biosynthesis [KO00900], Monoterpenoid biosynthesis [KO00902], phenylalanine, tyrosine, and tryptophan [KO00400], and indole alkaloid biosynthesis [KO00901]. Specifically, these pathways encompassed 2, 3, 1, and 5 genes encoding enzymes respectively. Notably, these enzymes are encoded by multiple genes, and the meta-analysis has demonstrated its ability to detect more genes compared to individual studies [29,30].

**Fig. 7:**
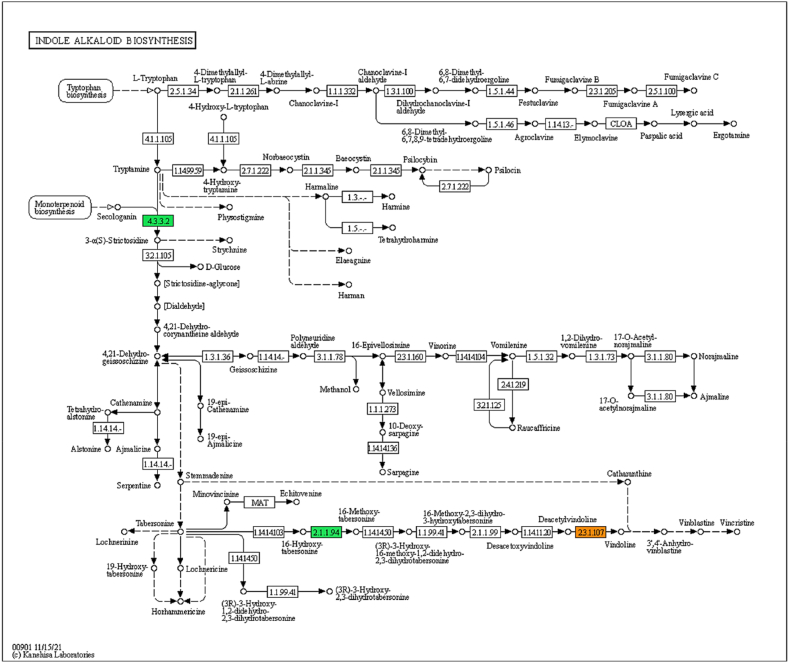
The DEGs related to the indole alkaloid biosynthesis pathway in *C. roseus* under hormone influence, indicating the enzymes encoded by *meta*-DEGs through colored rectangles.

Regarding the TIAs pathways, the meta-analysis identified 9 enzymes encoded by the DEGs. Fig. 7 illustrates the Indole alkaloid biosynthesis pathway, which represents the final pathway contributing to the production of vincristine and vinblastine. Additionally, other pathways are depicted in Fig. S2.

### 3.4 Transcription factors (TFs), protein kinases (PKs), and potential miRNAs detection

**Fig. 8:**
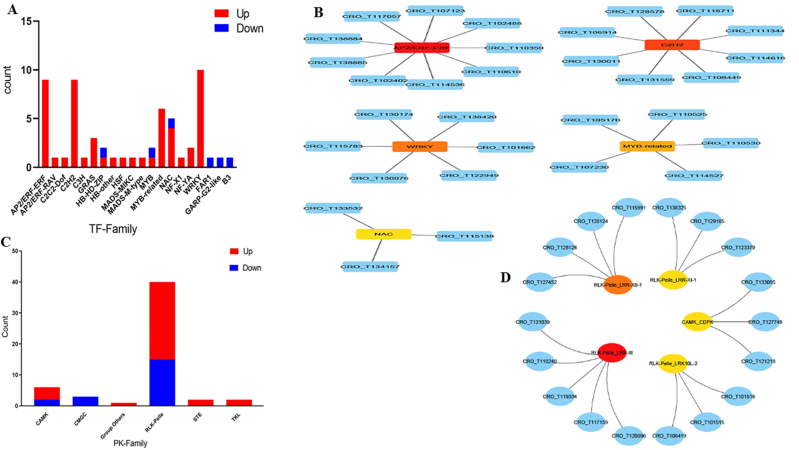
The Frequency occurrence of PK and TF families within the DEGs and interactions among DEGs-TFs/PKs. (A) The number of genes in TF families, and (B) the interactions among DEGs-TFs, (C) the number of genes in PK families, (D) the interactions among DEGs-PKs. Additionally, panels A and C also show the up- and down-regulation of TFs and PKs, and the top 5 identified TFs are depicted in panels B and D.

Through regulating transcription, transcription factors (TFs) wield significant influence over plant stress responses and the synthesis of secondary metabolites. Upon aligning DEG sequences with the iTAK database, we uncovered genes encoding transcription factors. These consisted of a total of 59 TF genes spanning twenty distinct TF families. Prominent classes included representatives from the WRKY, AP2/ERF, C2H2, and MYB-related families. Notably, six TFs showed downregulation, while 53 TFs exhibited upregulation overall. An intriguing observation was the complete downregulation of all members within the FAR1, GARP-G2-like, and B3 families, contrasted with the substantial upregulation of every member within the WRKY, AP2/ERF, and C2H2 families (Fig. 8A, Table S5). Additionally, Fig. 8B displays the network depicting interactions among DEGs-TFs, highlighting the top 5 identified TFs.

Plants employ protein kinases (PKs) as regulators for signaling responses to environmental stresses (Ling Ho, 2015). Among the DEGs, we uncovered 54 PK genes, classified into families including CAMK, CMGC, Group Others, RLK-Pelle, STE, and TKL. Notably, the RLK-Pelle family emerged as the most prevalent. Among these families, 34 PKs showed upregulation while 20 exhibited downregulation (Fig. 8C–Table S6). LRK10, identified as a potential receptor-like serine/threonine-protein kinase, displayed the lowest E value among the upregulated PKs. Furthermore, Fig. 8D illustrates the network illustrating interactions between DEGs-PKs and highlights the top 5 identified PKs.

We utilized MepmiRDB and psRNATarget (maximum expectation: 2.0) to predict potential miRNAs that could target the DEGs. Since the miRNAs in *C. roseus* are not fully characterized, we aligned the sequences with *Arabidopsis thaliana* miRNAs, given their well-established data and extensive research. From this analysis, MepmiRDB and psRNATarget identified a total of 12 and 96 miRNAs (maximum expectation: 2.0), respectively, belonging to 19 conserved families (Table S7). Among the identified miRNAs, MIR414, MIR169_1, ath-miR5021, and ath-miR5658 were the most prevalent, with 9, 7, 23, and 21 members, respectively. These miRNAs were found to target genes associated with the Plant high-mobility-group (*HMG*, *HMGB* gene), Nuclear transcription factor Y subunit A (*NFYA* gene), and CCR4-NOT transcription complex subunit 11 (*CNOT11*).

### 3.5 Promoter motif analysis of DEGs

We examined the regions flanking the DEGs, specifically 1000 base pairs upstream, in search of conserved patterns and consensus *cis*-regulatory elements (CREs). Employing MEME, we discovered ten noteworthy motifs within the DEG promoter, varying in length from 6 to 30 amino acids (Table 1). Notably, a significant portion of these identified motifs belonged to categories such as ERF/DREB, DOF, and BPC, associated respectively with AP2/EREBP, other C4 zinc finger transcription factors, and BBR/BPC (Table S8).

**Table 1:** The MEME analysis revealed conserved motifs in the DEG promoter.

Motif	Logo	P-value	Width	Best match in JASPAR	Family	Significant GO term identified by GOMO
Motif 1		3.05E-04	20	MA1738.1	Myb	–
Motif 2		2.92E-03	21	MA1008.1	ERF/DREB	CC chloroplast
						CC mitochondrion
Motif 3		1.03E-03	41	MA1351.2	ERF/DREB	CC mitochondrion
						CC chloroplast thylakoid membrane
Motif 4		9.25E-07	41	MA1403.1	BBR/BPC	MF transcription factor activity
						CC mitochondrion
						CC CUL4 RING ubiquitin ligase complex
						BP root hair cell tip growth
						BP regulation of transcription
Motif 5		1.47E-03	19	MA1740.1	bHLH	CC mitochondrion
						CC chloroplast
Motif 6		6.05E-05	14	MA1226.1	ERF/DREB	MF transcription factor activity
						CC chloroplast
Motif 7		1.17E-02	21	MA2045.1	NAC	CC chloroplast
Motif 8		3.30E-07	15	MA1274.1	DOF	MF transcription factor activity
						CC plasma membrane
Motif 9		5.71E-10	21	MA1274.1	DOF	CC nucleus
						CC plasma membrane
						BP regulation of transcription, DNA-dependent
						BP transmembrane receptor protein tyrosine kinase signaling pathway
Motif 10		1.22E-05	28	MA1404.1	BBR/BPC	MF transcription factor activity
						CC nucleus
						MF protein binding
						BP regulation of transcription
						MF RNA binding

The GOMO analysis conducted on the MEME motifs unveiled a numerous relevant biological function, as detailed in Table 1 and Table S9. As gene ontology findings, these motifs played roles in root hair cell tip growth, transcription regulation, DNA-dependent processes, and transmembrane receptor protein tyrosine kinase signaling pathways (Table S9). Moreover, these motifs were found to be associated with molecular functions including transcription factor activity, protein binding, and RNA binding (Table 1).

### 3.6 Co-expression analysis

**Fig. 9:**
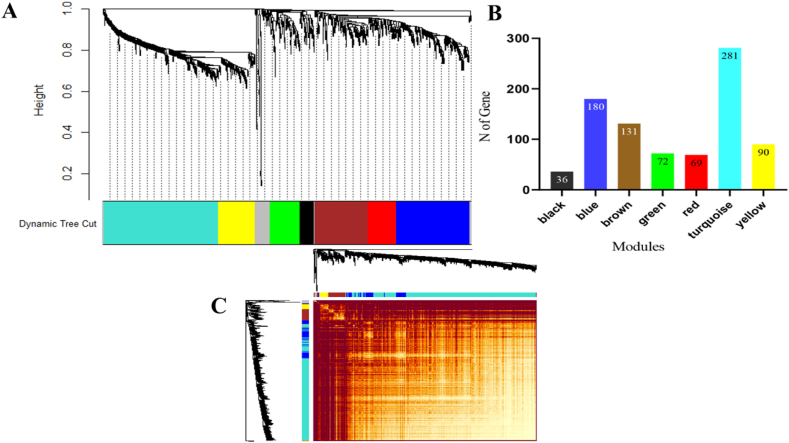
The weighted gene co-expression network analysis. (A) The hierarchical cluster tree of the DEGs, (B) the gene count within each module and (C) the heatmap plot of the network's topological overlap matrix (TOM).

Employing a systems biology approach, WGCNA was utilized to identify the DEGs with the strongest correlations among them. Employing the dynamic tree cutting algorithm, the DEGs were clustered into seven modules, each containing a range of 36–281 genes (Fig. 9A and B). Notably, three primary modules stood out: turquoise (281 genes), blue (180 genes), and brown (131 genes). Fig. 9C illustrates the topological overlap matrix (TOM) generated from the hierarchical clustering of the DEGs. An analysis focusing on the biological process category was conducted to grasp the biological functions related to the modules. Within the turquoise module, a significant enrichment was observed in the GO term related to RNA modification. Meanwhile, the blue and green modules were linked to the regulation of transcription, DNA-templated. The brown, black, red, and yellow modules were associated with defense response, response to water deprivation, protein ubiquitination, and embryo development ending in seed dormancy, respectively.

Furthermore, within the green module, crucial GO terms significantly associated to hormone response included response to salicylic acid, cellular reaction to jasmonic acid stimulus, hormone-mediated signaling pathways, while the red module exhibited enrichment in regulating the salicylic acid biosynthetic process. Additionally, the blue, turquoise, and green modules harbored the highest number of genes associated with the biosynthesis pathway of TIAs. Comprehensive details on the enrichment analysis results for the modules are available in Table S10.

### 3.7 Hub gene investigation within modules

**Fig. 10:**
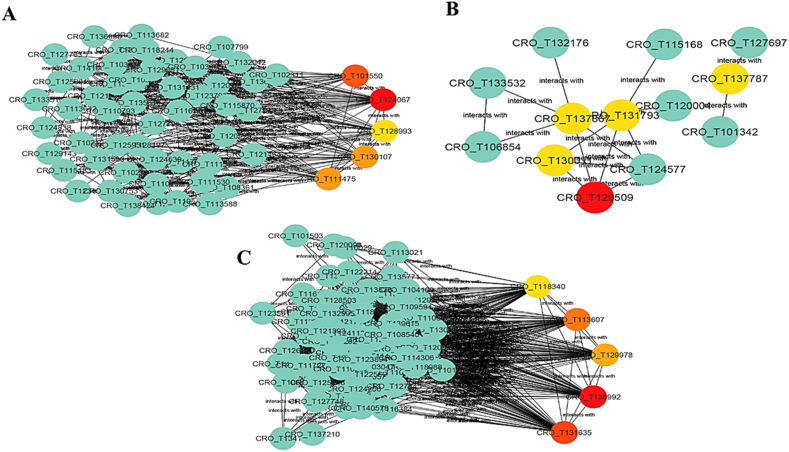
The hub genes and intramodular connections within the blue (A), green (B), and turquoise (C) modules. (For interpretation of the references to color in this figure legend, the reader is referred to the Web version of this article.)

The identification of hub genes within each module highlighted genes with pivotal roles in the network, indicating their crucial functionalities. Within every module, five hub genes were discerned (Table S11). These hub genes exhibited substantial enrichment in responses to oxidative stress, metal ion binding, and protein binding. Notably, genes like *hbdA* (*CRO_T136992*) and *Acy1b* (*CRO_T131635*) displayed the highest connectivity within the network. Results further showcased ALP1 (CRO_T129509) within the green module as possessing the highest degree of connectivity between two modules associated with hormone responses. Moreover, the blue, turquoise, and green modules contained a significant number of genes related to the TIAs biosynthetic pathway. Additionally, apart from *hbdA* and *Acy1b* in the turquoise module and the *ALP1* gene in the green module, *IGHMBP2* (*CRO_T124067*), and *SIGE* (*CRO_T101550*) within the blue module exhibited the highest connection scores in the network. Fig. 10 visually presents the hub genes and intramodular connections within the blue, green, and turquoise modules.

### 3.8 Verification using RT-qPCR

**Fig. 11:**
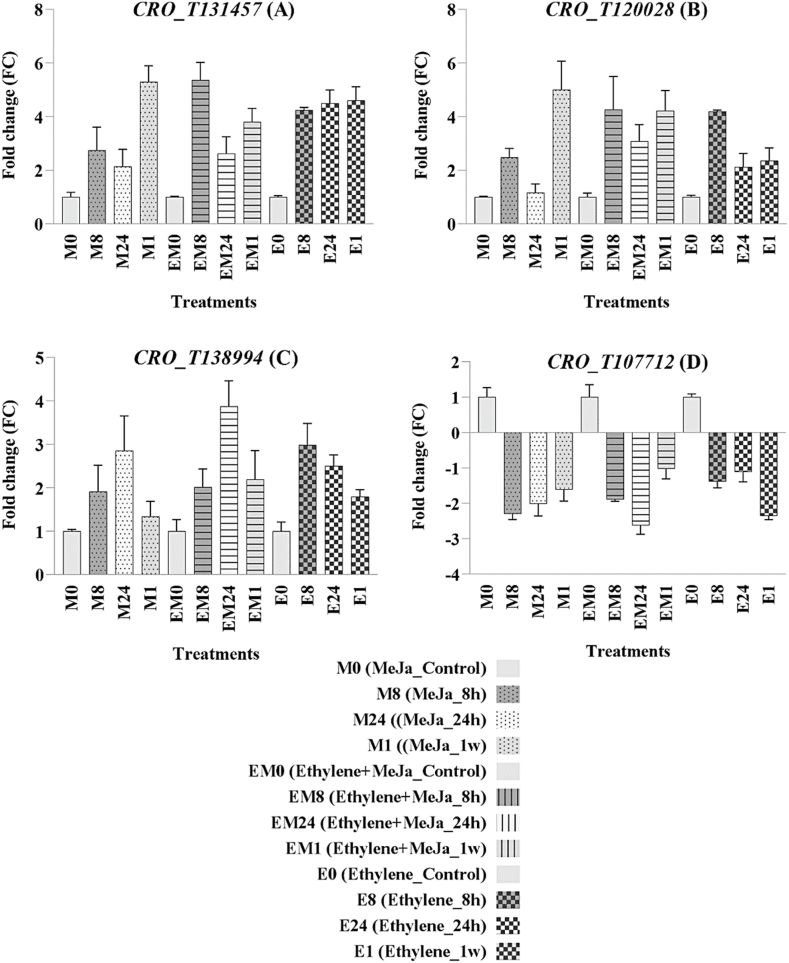
The relative expression levels of four chosen genes: (A) *CRO_T131457*, (B) *CRO_T120028*, (C) *CRO_T138994*, and (D) *CRO_T107712*.

RT-qPCR experiments were conducted on *C. roseus* plants subjected to methyl jasmonate, ethylene, and a combination of both hormones to validate the findings of the meta-analysis. Four specific genes identified from the meta-analysis (*CRO_T131457*, *CRO_T107712*, *CRO_T138994*, *CRO_T120028*), potentially associated with the biosynthesis of TIAs responsible for producing the valuable anticancer compounds vincristine and vinblastine, were selected. The expression of each gene was compared between treated and control plants (Fig. 11). The results indicated a correlation between the expression patterns of these chosen *meta*-DEGs under treatment conditions and the findings from the meta-analysis. These findings substantiate the accuracy and reliability of the meta-analysis results.

## 4 Discussion

### 4.1 Identified DEGs, Gene ontology and pathway enrichment

*C. roseus* stands as a vital medicinal plant, serving as the exclusive origin of various TIAs, notably including the anticancer compounds vincristine and vinblastine. MeJA and ethylene hormones act as essential factors, known as abiotic elicitors, promoting the biosynthesis and augmentation of specific secondary metabolites in medicinal plants, as highlighted in research by Singh et al. (2018), and Rahmat and Kang (2019) [2,3]. These hormones are key signals and factors coordinating plant responses and stress reactions. Multiple pathways regulate plant responses to these factors, often interconnecting through the activation of transcription factors and protein kinases, pivotal regulators in signaling. Understanding the intricacies of these processes governing plant responses to these stimuli holds paramount importance. Thus, we combined transcriptomic data from *C. roseus* treated with two distinct hormones via a meta-analysis approach to unveil critical genes and pathways. To our knowledge, this study marks the first effort to conduct and present a meta-analysis encompassing multiple RNA-Seq datasets stimulated by various hormones in this plant species. This innovative method enabled the identification of genes pivotal in response to different hormones, serving as elicitors, and facilitated the recognition of a set of abiotic stress-related components. For this purpose, we compiled RNA-seq data from 17 samples of *C. roseus* sourced from publicly available datasets, focusing on two distinct exogenous hormones (Table S3). The meta-analysis unveiled a total of 903 DEGs (Table S4), among which 59.14% exhibited upregulation while 40.86% displayed downregulation. This discovery emphasizes the heightened activity of diverse biological processes, including those recognized as intermediary signaling molecules crucial in mediating numerous biological pathways. These alterations likely contribute to the accumulation of elicitor-induced TIAs [31].

Previous studies investigating *C. roseus* have identified DEGs. For instance, Pathania et al. (2016) characterized 858 genes to pinpoint those linked to metabolite production [32]. In another study by Pan et al. (2018), 383 and 1095 genes exhibited different expression levels in response to ET or MeJA, with 1214 showing upregulation and 264 displaying downregulation [5]. The meta-analysis we conducted unveiled novel genes that were not identified in individual studies, even though the total count of these genes in our study might not surpass the quantity found in those studies.

The functional enrichment analysis highlighted diverse biological functions enriched among the DEGs (Fig. 5). Key terms such as Cellular process, Metabolic process, Cellular metabolic process, Organic substance metabolic process, and Response to stimulus emerged as significant, indicating the involvement of numerous genes and enzymes in metabolite efflux. Plants adapt their metabolism genetically in response to various stimuli and stresses, influencing metabolite synthesis. They adeptly respond to these conditions by engaging various metabolic pathways, thereby synthesizing secondary metabolites. Specific metabolic pathways exhibiting upregulation of proteins and metabolites are believed to underlie enhanced defensive responses during stressful situations and stimuli [33].

The enhancement of tolerance in *C. roseus* and other plant species might correlate with the up-regulation of specific metabolic pathways triggered by increased stress levels. Transcriptome analysis unveiled a set of DEGs implicated in the regulation of defense responses, potentially influencing stress response mechanisms and regulate secondary metabolism. Consistently, preceding studies highlighted primary biological process categories encompassing cellular process, metabolic process, biological regulation, and response to stimulus terms [[34], [35], [36], [37]]. Certain genes, such as Germin-like protein subfamily T member 1 (*GLP1*), Monoterpene synthase 7 (*TPS7*), alpha-terpineol synthase (*ATESY_VITVI*), and Pathogenesis-related genes transcriptional activator (*PTI5*), exhibited notably upregulated expression levels. Research has demonstrated the involvement of *'GLPs'* in plant reactions to diverse stresses and defensive responses. Furthermore, *GLPs* have demonstrated roles in cell wall remodeling or enzyme activity, as well as being structural proteins and receptors [38].

Past studies have highlighted the significance of TPSs across diverse biological processes, underscoring their pivotal role as primary enzymes in terpene biosynthesis [[39], [40], [41]]. Alpha-Terpineol, a monoterpenoid compound found in plants, operates as a crucial plant metabolite [42]. Additionally, *Pti5* is categorized within the ethylene-response factors (ERFs) family, known for their involvement in defense responses via the regulation of pathogenesis-related genes (*PRs*) expression. Sun et al. (2022) identified *Pti5's* involvement in overseeing the signaling pathways of JA, SA, and ethylene [43]. Falak et al. (2021) observed that overexpressing PTIs in *Arabidopsis* triggered a defensive response and increased tolerance against *P. syringae*. Furthermore, it was found that numerous DEGs that are related to metabolic processes and responses to stimuli, including Ethylene-responsive, WRKY, and MYBs transcription factors [44]. These findings potentially indicate a connection with the regulation of secondary metabolism and stress responses.

Pathway enrichment analysis of the DEGs highlighted crucial metabolic pathways (Fig. 6). The extensive presence of genes involved in metabolic pathways, particularly in secondary metabolite synthesis, has been linked to substantial adjustments in plant metabolism as part of stress adaptation. Secondary metabolites serve defensive roles against stresses and are pivotal in regulating such challenges [45]. Notably, the Starch and sucrose metabolism pathway also exhibited enrichment. Starch serves as the primary energy source for plants and plays a role in stress response mechanisms. Intermediate products within this pathway might potentially interact with other signaling pathways to modulate downstream responses, such as osmotic regulators and signaling molecules [46]. Apart from these pathways, our analysis revealed the enrichment of glycolysis/gluconeogenesis, indicating its potential involvement in stress responses. Previous studies have demonstrated that stress induces changes in the expression of key enzymes associated to glycolysis/gluconeogenesis metabolism [47]. The glycolysis process involves the conversion of glucose (derived from starch and sucrose) into pyruvate. Subsequently, the TCA cycle decarboxylates this pyruvate to generate reductants [48]. Various intermediates within the TCA cycle play roles in the synthesis of several alkaloids [49]. Furthermore, additional pathways such as Purine, cysteine, and methionine metabolism; Valine, leucine, and isoleucine degradation; and Glycine, serine, and threonine metabolism also exhibited enrichment, underscoring their potential significance in confronting stress [50,51].

Regarding the TIA pathway genes among the DEGs, our analysis identified the involvement of 3 enzymes and 5 genes within the Indole alkaloid biosynthesis pathway. Additionally, we found 1, 2, and 3 enzymes, along with 1, 2, and 3 genes related to pathways of “phenylalanine, tyrosine, and tryptophan biosynthesis”, “terpenoid backbone biosynthesis”, and “monoterpenoid biosynthesis”, respectively (Fig. 7, Fig. S2). These enzymes are encoded by multiple genes, and the meta-analysis has notably revealed a higher number of genes compared to individual studies [29,30].

### 4.2 Identification of TFs, PKs and miRNAs

TFs serve as critical molecular regulators pivotal for gene transcription, enhancing plants' adaptability and responsiveness to various stimuli. Understanding how genes regulate in *C. roseus* under such conditions is crucial. The DEGs comprised 59 TFs spanning 20 TF families (Table S5). Prominent families among these TFs include WRKY, AP2/ERF, C2H2, and MYB. Notably, in these conditions, significant members of the WRKY, AP2/ERF, and C2H2 TF families exhibited upregulation, whereas key members of the FAR1, GARP-G2-like, and B3 families were observed to be downregulated (Fig. 8A). The TFs AP2/ERF, WRKY, C2H2, bHLH, and MYB-related are key regulators of alkaloid biosynthesis pathways in *C. roseus* and various other plant species [52]. Within plants, the AP2/ERF family regulates secondary metabolites and stress responses. Studies have demonstrated that upregulating ERF family genes enhances metabolite production across diverse plant species like birch, rice, and *C. roseus* [[53], [54], [55]]. Notably, the overexpression of AP2/ERF resulted in a significant increase in MIA content in *C. roseus*, consequently elevating alkaloid accumulation in the plants [54]. Meanwhile, the WRKY TF family assumes a vital role in plant stress responses. In our study, all 13 WRKY members showed upregulation. In *C. roseus*, CrWRKY1 and CrMYC1 have been linked to vincristine alkaloid production. The overexpression of CrWRKY1, a MeJA-responsive WRKY gene in *C. roseus*, suggests its potential role in regulating the TIA biosynthesis pathway [56,57].

Six MYB-related TFs and all nine C2H2-related TFs exhibited upregulation. Studies have revealed that various C2H2 zinc finger proteins elevate plant stress tolerance by activating comparable ABA or protein kinase signaling pathways [58]. MYB-related TFs encompass a range of functions, including the regulation of secondary metabolite production, developmental processes, and plant responses to stresses [59]. While numerous MYB TFs have been identified as regulators of specialized metabolite production [60], only a few have been implicated in the regulation of alkaloid biosynthesis [57]. *CrBPF-1*, a gene in *C. roseus*, encodes a MYB-like box-P binding factor protein containing an elicitor responsive element [56,57].

FAR1, GARP-G2-like, and B3 exhibited downregulation, each with a count of one, signaling a reduction in their expression levels. Research has shown that FAR1 suppresses plant growth by directly stimulating the expression of two unusual bHLH transcriptional cofactors, a process countered by a set of JASMONATE ZIM-DOMAIN proteins. These proteins serve as key repressors within the jasmonic acid (JA) signaling pathway [61]. GARP transcription factors assume crucial functions in plant growth and their reaction to environmental stimuli. For instance, *GARP's NIGT1* genes respond to phosphorus (P) and nitrogen (N) stresses, with some being downregulated following nitrogen stresses (NS) treatment [62]. Additionally, B3 subfamily proteins within the ERF family are associated to the regulation of secondary metabolites [63]. As a consequence, previous investigation of TF expression validates our observations regarding their regulatory involvement in stress responses and the synthesis of secondary metabolites.

We explored potential protein kinases responsible for post-translational regulation of target proteins and signaling networks, aiming to delve into signal transduction processes in *C. roseus* under stress and stimulation. Within the DEGs, we identified 54 PKs categorized into RLK Pelle, CAMK, CMGC, STE, TKL, and Group Others (Table S6, Fig. 8C). Notably, among the upregulated PKs, LRK10, a likely receptor-like serine/threonine-protein kinase, exhibited the lowest E-value in stress conditions. LRK10L serves as a positive regulator in certain stress responses and is associated with ABA-mediated signaling [64]. This gene belongs to the Ser/Thr protein kinase family within the RLK/Pelle class of protein kinases superfamily. Under stimulation conditions, RLK-Pelle was also observed to be upregulated [65]. RLKs are pivotal in signaling pathways that confer resistance to various stresses and strengthen plant defense against pathogen intrusion by activating immune responses. Expression data highlights the significance of RLK/Pelles in responding to stress. Studies indicate that numerous RLK/Pelles exhibit upregulation during stress, potentially impacting metabolite production through the activation of the plant's defense system against stimuli. Moreover, the extent of tandem duplication within RLK/Pelle subfamilies is related to stress responsiveness [66,67]. RLKs play vital roles in regulating plant development, signaling networks, disease resistance, defense mechanisms and responding to a range of stresses [[68], [69], [70]]. The CrRLK1L receptor kinase is pivotal in governing plant growth, development, and stress responses. Presently, Arabidopsis and rice genomes have identified 17 and 20 putative CrRLK1L members, respectively, with some of their functions elucidated [71]. Interestingly, while all genes in the CMGC and TKL families displayed downregulation, the RLK-Pelle class exhibited upregulation. Consequently, our findings suggest that stress and stimulation significantly impact various protein kinase types, indicating both positive and negative interactions among protein kinases.

A vast families of non-coding RNAs, commonly known as miRNAs, actively participates in diverse biological functions, including stress responses and plant hormone homeostasis. Recent investigations have begun uncovering their involvement in controlling the production and regulation of secondary metabolites, although this understanding remains limited. The specific role of miRNAs in regulating TIA biosynthesis in *C. roseus* has not been well studied. Existing studies indicate that miRNAs play roles in fiber development, stress responses, and various biochemical pathways in this species [72,73]. In our current study, a total of 12 and 96 miRNAs were identified using MepmiRDB and psRNATarget, respectively, all belonging to 19 conserved families (Table S7), many of which are implicated in multiple biological processes. The number of miRNAs identified in our research differs from those reported in other studies, for instance, 181 conserved and 173 novel miRNAs noted by Shen et al. (2017), and 88 potential miRNAs categorized within 35 families in *C. roseus* according to Prakash et al. (2015) [72,73]. In our study, we applied a stringent criterion of maximum expectation: 2.0, significantly reducing the count of miRNAs. Most of the miRNAs identified in our study belong to conserved families, including miR414, miR169_1, and ath-miR5021. It has been revealed that miR414 targets genes involved in signal transduction, metabolism, plant growth, and development, namely the Plant high-mobility-group (*HMG*, *HMGB* gene), Nuclear transcription factor Y subunit A (*NFYA* gene) (*NF-YA* gene family members), and CCR4-NOT transcription complex subunit 11 (*CNOT11*) [74]. MiR414 has been identified as a regulator of triterpenoid biosynthesis in previous research [75]. In rice, the miRNA Osa-MIR414 targets the *OsABP* gene, and during salt stress, the target genes show an up-regulated pattern while the miRNAs demonstrate down-regulation [76]. The miR169 family has shown significant importance in various plant species like tomato, rice, and banana in their responses to stresses, as indicated in prior studies [[77], [78], [79], [80]]. Additionally, ath-miR5021 participates in the regulation of transcripts related to diverse processes such as DNA recombination, repair, nucleosome assembly, among others. In the *Arabidopsis thaliana*, ath-miR5021 has been observed to collaborate with several miRNA families within studies exploring the microRNA-microRNA crosstalk network. Interestingly, in the same investigation, ath-miR5021, ath-miR5658, and ath-miR414 displayed the highest levels of network connectivity, establishing links with other miRNA families, a finding that supports our meta-analysis. This observation suggests that these highly-connected miRNAs act as crucial nodes connecting different clusters within the network [81]. Therefore, the outcomes from prior studies corroborate and validate the results obtained from our miRNA prediction analysis, largely supporting them. Consequently, these identified miRNAs possess the potential to target genes associated with stress response or metabolite synthesis. In particular, regarding TIAs, these miRNAs could serve as potential candidates for future investigations.

### 4.3 Identification of motifs

Promoter analysis was utilized to detect specific *cis*-acting elements regulated by common upstream regulators of DEGs. A set of ten motifs with significant scores was identified (Table S8). There's a probable interaction between plant stress responses and various *cis*-regulatory components. Our findings included binding motifs associated with ERF/DREB (AP2/EREBP) (motifs 2, 3, 6, and 9), BBR/BPC (motifs 2 and 10), DOF (Other C4 zinc finger-type factors) (motifs 8 and 9), Myb (Tryptophan cluster factors) (motif 1), Basic helix-loop-helix factors (bHLH) (motif 5), and NAC (GCM domain factors) (motif 7) transcription factor families. The motifs show a strong corresponding to the ERF/DREBs (Table 1), a prominent group of plant-specific TFs known for their highly conserved AP2/ERF DNA binding domain. AP2/ERFs have become pivotal in plant metabolic regulatory networks, responding to ABA and ET hormones to enhance plant survival under stressful conditions [82]. Numerous AP2/ERFs have been identified as crucial in JA-responsive signaling, especially in regulating MIA biosynthesis, with the MeJA-responsive group IX ERF subfamily members playing a significant role in this process [57,60].

### 4.4 Identification of Co-expression modules and hub genes

A co-expression network analysis of the DEGs was conducted to uncover the processes related to abiotic stimulation. Seven key functional clusters, represented as modules (Fig. 9), were identified. Through module enrichment analysis (Table S10), relationships among various biological processes such as transcriptional regulation, stress and defense responses were revealed within these modules. Notably, the green and red modules displayed a high correlation with responses to hormones and stimuli. Upon closer investigation, it was observed that these modules contained 19 genes linked to hormonal responses and stimuli, while 114 genes were predicted to regulate diverse biological processes. Furthermore, we identified five pivotal hub genes within each module. In the turquoise module, *hbdA* (*CRO_T136992*) and *Acy1b* (*CRO_T131635*) displayed the highest connectivity within the module's network (Table S11, Fig. 10). Notably, the blue, turquoise, and green modules showcased a notable association with TIAs pathways, harboring 6, 4, and 3 genes, respectively. Among the five hub genes within these modules, the Helicase ATP-binding domain-containing protein, *hbdA*, and *ALP1* genes demonstrated the strongest connections in the networks of the blue, turquoise, and green modules, respectively. For instance, 3-hydroxybutyryl-CoA dehydrogenase (*hbdA*) functions as an NADH-specific enzyme, yet its precise roles and applications remain relatively unclear [83]. Additionally, aminoacylase-1 (*ACY-1*) has been implicated in contributing to *Nicotiana benthamiana*'s resistance against pathogen infections. However, its role in plant growth and responses to environmental stress remains poorly understood. *ZmACY-1* has been indicated to contribute to plant breeding efforts under abiotic stress conditions, playing a pivotal role in both plant growth and defense mechanisms [84]. ALPs, classified within the PIF/Harbinger superfamily of transposons, include genes like *ALP1* (*CRO_T129509*), which demonstrated a higher score within the green module. Mutations in *ALP* genes have resulted in a mild late-flowering phenotype, suggesting their involvement in the process of floral induction [85]. RNA helicases serve critical functions in gene expression regulation and can be influenced in response to specific environmental changes. For instance, abiotic stress has been observed to impact the rice DEAD-box helicase ATP-binding protein (*OsABP*) [76].

We determined pivotal genes with significant connectivity within and between meta-modules, specifically enriched for stress response mechanisms and the synthesis of secondary metabolites. These hub genes, identified within stress-responsive and metabolite-related modules, likely play essential roles in these conditions, displaying strong correlations with other genes. The reported co-expression relationships can be a valuable resource for inferring potential functions of genes encoding proteins with yet unknown activities in *C. roseus*. In our investigation, we unearthed certain genes within *C*. *roseus* that have not been previously explored or documented in this species. We identified these genes through diverse methods, aligning their sequences with known genes, suggesting their potential significance in regulating and synthesizing secondary metabolites. The primary drawback of our study lay in the limited number of samples due to the constraints of available RNA-Seq studies that met our criteria up to the date of our research. This constraint may have hindered a comprehensive understanding of the species' response to stress. Consequently, to strengthen our confidence in the identified genes and regulators resulting from the meta-analysis, we conducted various downstream analyses. To validate our findings further and represent enriched metagens in the TIAs biosynthetic pathway, we confirmed the expression of four genes using RT-qPCR.

The gene *CRO_T120028* exhibited homology with several genes involved in the TIA biosynthesis pathway, with the most significant one being *DAT*. This gene in the *C. roseus* plant encodes the enzyme deacetylvindoline-4-*O*-acetyltransferase, which is involved in the final stages of vindoline biosynthesis. The results obtained in this study were consistent with previous studies wherein the application of various stress treatments led to an increase in *DAT* expression and consequently enhanced production of important medicinal alkaloids in *C. roseus* [6,[86], [87], [88], [89]].

The gene *CRO_T131457* showed homology with the *tryptophan synthase* gene (*TSB*), which is involved in the biosynthesis pathway of phenylalanine, tyrosine, and tryptophan in the TIAs biosynthesis pathway. In the current study, this gene demonstrated upregulation in response to ethylene and methyl jasmonate treatments. Similarly, in Arabidopsis, the expression of this gene has been confirmed to be influenced by hormones as well as various stresses [[90], [91], [92], [93]].

The gene *CRO_T107712* showed homology with the *AACT* gene, which encodes for the enzyme acetyl-CoA C-acetyltransferase involved in the biosynthesis pathway of TIAs. This enzyme catalyzes the combined reaction of two acetyl-CoA molecules to form acetoacetyl-CoA, representing the initial enzymatic step in the mevalonate (MVA) biosynthesis pathway. The results were not consistent with the majority of previous studies regarding the gene *AACT*, which have observed upregulation of the *AACT* gene under different treatments. This inconsistency could potentially arise from variations in the treatments applied, their concentrations, and the plant species under investigation. Studies specifically focusing on the effect of this gene on stress tolerance and secondary metabolite production in *C. roseus* were lacking, and limited studies were found on other plant species as well [81,[94], [95], [96], [97], [98]].

The gene *CRO_T138994* exhibited homology with the gene *CYP76A26*, which encodes the enzyme nepetalactol monooxygenase (previously described as iridoid oxidase) involved in the biosynthesis pathway TIAs. Studies investigating the expression of this gene under stress conditions and its role in metabolite production have been extremely limited and received less research attention, to the extent that no relevant studies were found through a search. Therefore, research on this gene in relation to stress responses and metabolite production appears to be a valuable and important endeavor.

## 5 Conclusion

In this study, a computational approach using systems biology and meta-analysis was applied to explore responsive genes within RNA-Seq data, aiming to comprehend how *C. roseus* reacts to plant hormones. The majority of the identified differentially expressed genes (DEGs) were associated with cellular and metabolic processes. Through promoter analysis, ten prominent *cis*-acting elements were identified in the upstream regions of these DEGs. The study further delves into the involvement of various transcription factors (such as WRKY, AP2/ERF, and C2H2), kinases (like serine/threonine-protein kinase), and miRNAs (including MIR414, MIR169_1, and ath-miR5021), all of which play crucial roles in hormone response mechanisms. Furthermore, the investigation revealed certain genes whose functions are not yet well understood. The network analysis identified hub genes within the network of three modules, including *hbdA* and *ALP1*, which might be involved in the pathways related to TIAs. The enrichment analysis of DEGs and biosynthetic pathways indicated the presence of several genes associated with TIA pathways, suggesting that the RNA-Seq meta-analysis under MeJa and ethylene hormones could potentially stimulate the expression of genes linked to the production of vinblastine and vincristine. These identified DEGs could serve as valuable resources for molecular exploration and as potential gene candidates for genetic and metabolic engineering studies in *C. roseus*.

## CRediT authorship contribution statement

**Seyede Nasim Tabatabaeipour:** Conceptualization, Data curation, Formal analysis, Validation, Writing – original draft. **Behrouz Shiran:** Conceptualization, Data curation, Supervision, Writing – review & editing. **Rudabeh Ravash:** Conceptualization, Supervision, Writing – review & editing. **Ali Niazi:** Supervision, Writing – review & editing. **Esmaeil Ebrahimie:** Conceptualization, Formal analysis, Supervision, Writing – review & editing.

## Declaration of competing interest

The authors declare that they have no known competing financial interests or personal relationships that could have appeared to influence the work reported in this paper.
