# Supplementary material for: Comprehensive transcriptomic meta-analysis unveils new responsive genes to methyl jasmonate and ethylene in Catharanthusroseus
Source: Heliyon. 2024 Feb 26;10(5):e27132. doi: 10.1016/j.heliyon.2024.e27132 (PMC10915408; doi:10.1016/j.heliyon.2024.e27132)
Supplement: Multimedia component 1 [file mmc1.docx]

(A) (B)


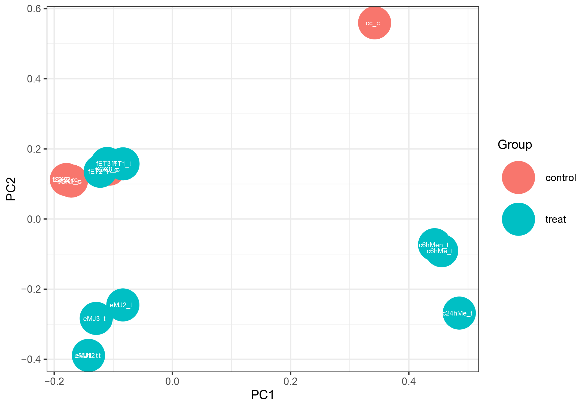

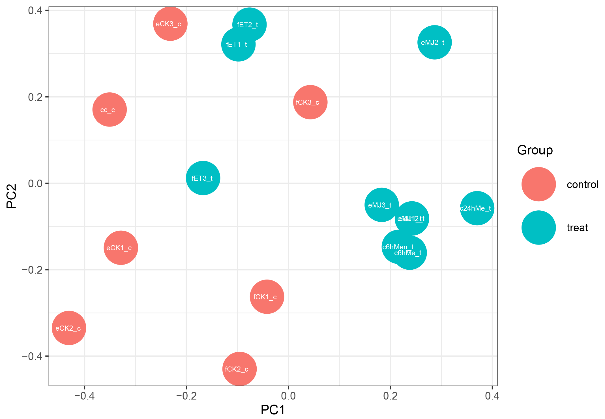


(C) (D)


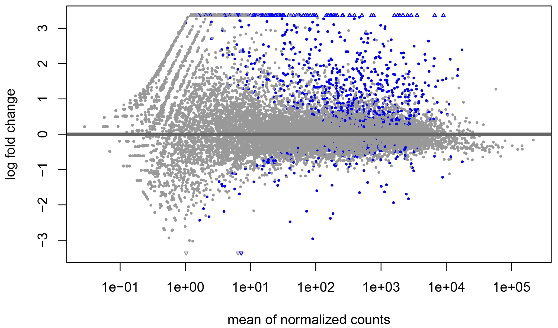

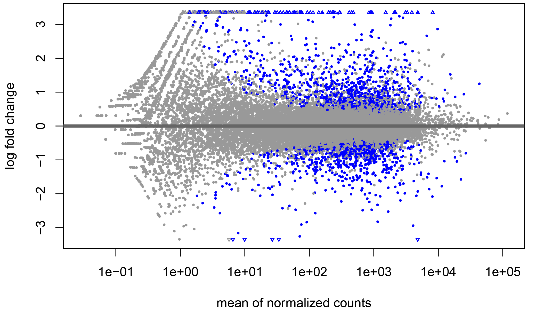


**Fig. S1.** Principle component analysis (PCA) and MA plot to assess the batch effect correction. (A) PCA before batch effect correction. (B) PCA after batch effect correction. (C) MA before batch effect correction. (D) MA after batch effect correction.
