# Supplementary material for: Comprehensive transcriptomic meta-analysis unveils new responsive genes to methyl jasmonate and ethylene in Catharanthusroseus
Source: Heliyon. 2024 Feb 26;10(5):e27132. doi: 10.1016/j.heliyon.2024.e27132 (PMC10915408; doi:10.1016/j.heliyon.2024.e27132)
Supplement: Multimedia component 2 [file mmc2.docx]

(A)


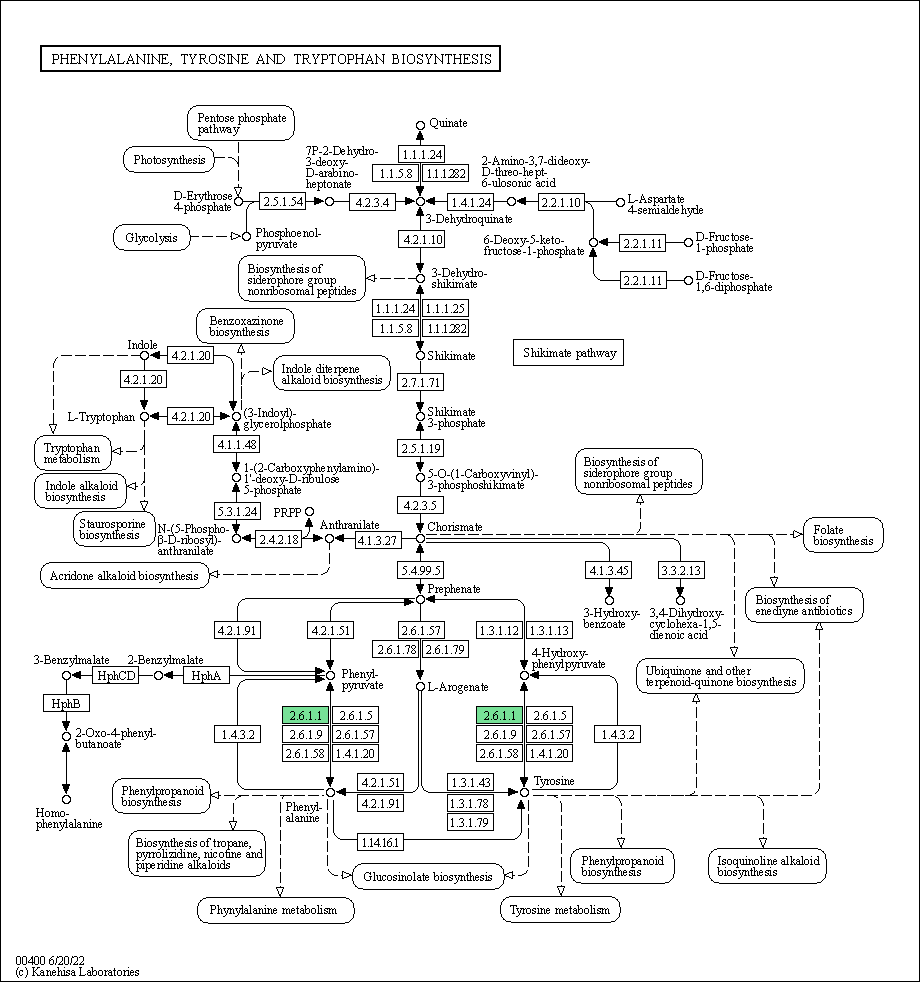


(B)


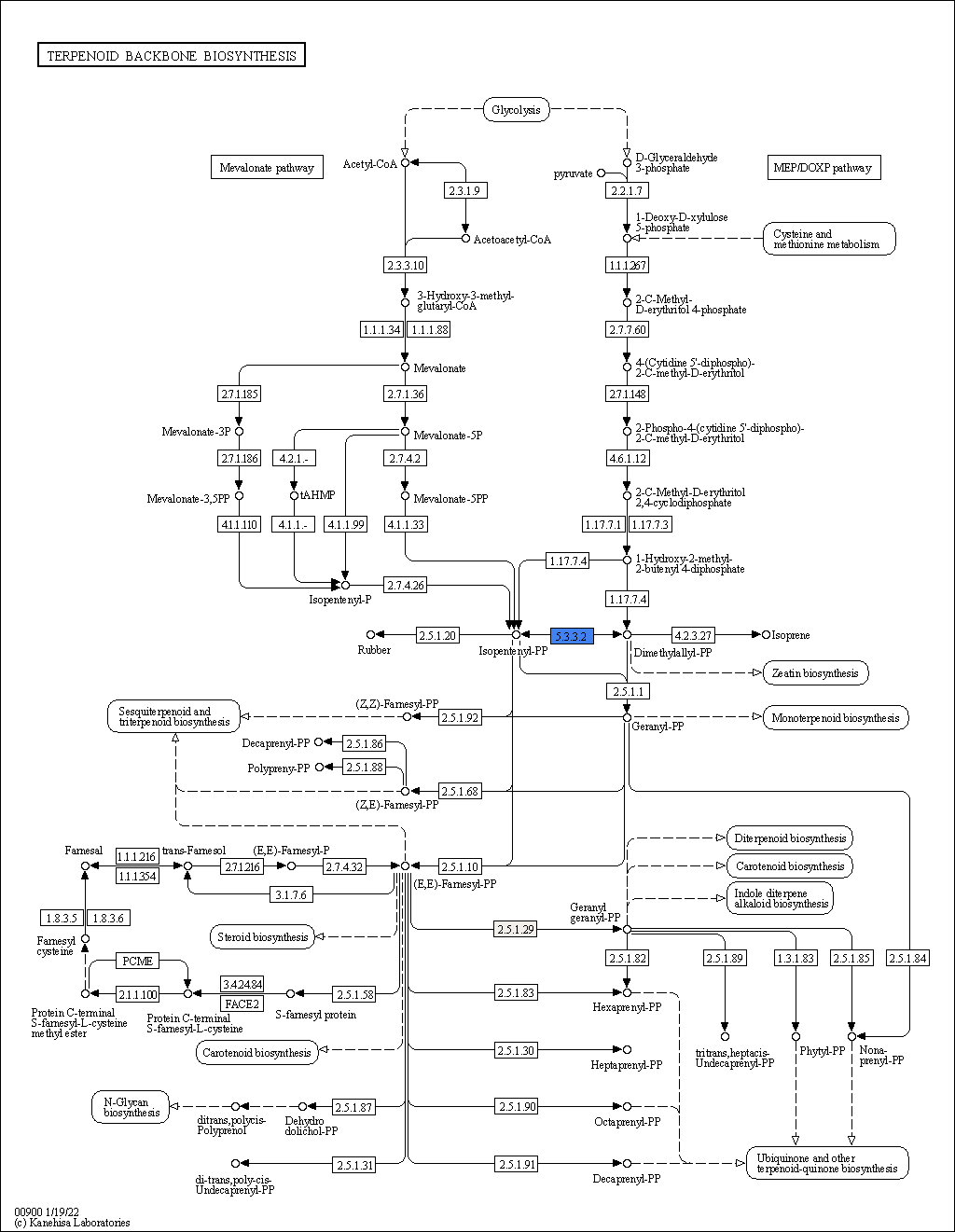


(C)


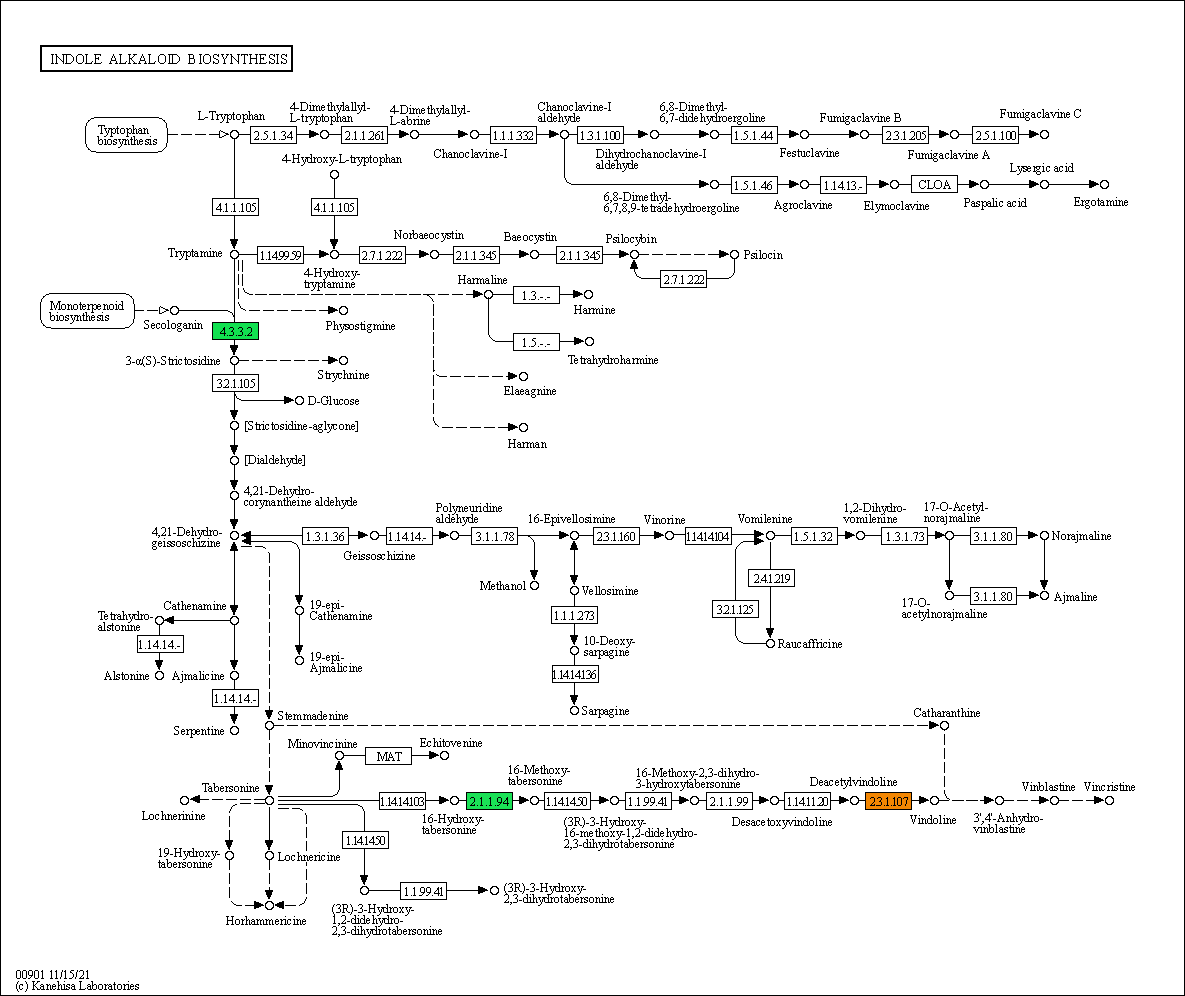


(D)


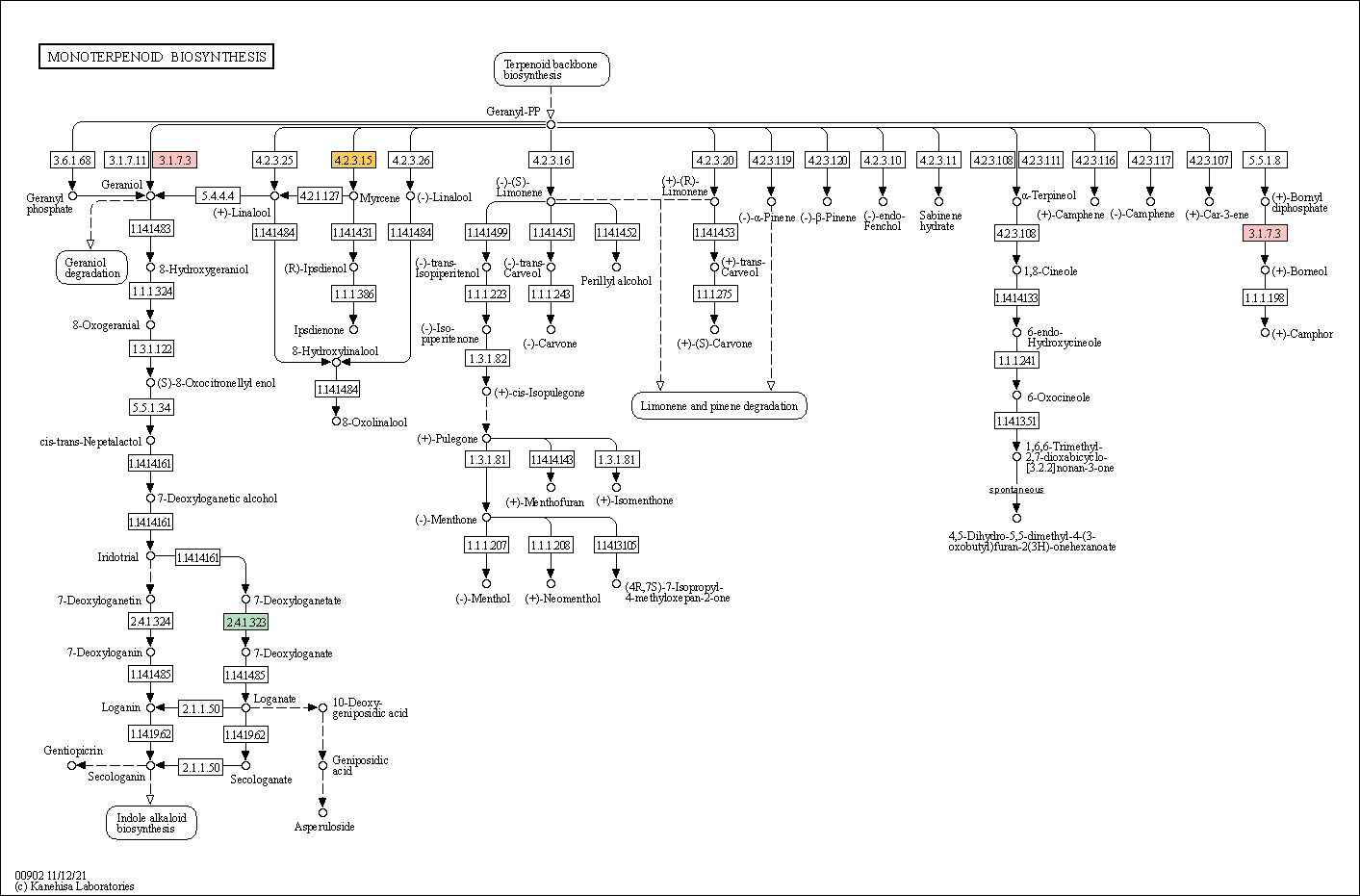


**Fig. S2.** DEGs related to indole alkaloid biosynthesis pathway in *C. roseus* in response to hormones. (A) Phenylalanine tyrosine and tryptophan biosynthesis, (B) Terpenoid backbone biosynthesis, (C) Indole alkaloid biosynthesis and (D) Monoterpenoid biosynthesis. Enzymes are encoded by meta-DEGs are shown by colored rectangles.
