# Supplementary material for: Comprehensive transcriptomic meta-analysis unveils new responsive genes to methyl jasmonate and ethylene in Catharanthusroseus
Source: Heliyon. 2024 Feb 26;10(5):e27132. doi: 10.1016/j.heliyon.2024.e27132 (PMC10915408; doi:10.1016/j.heliyon.2024.e27132)
Supplement: Multimedia component 3 [file mmc3.docx]

(A)


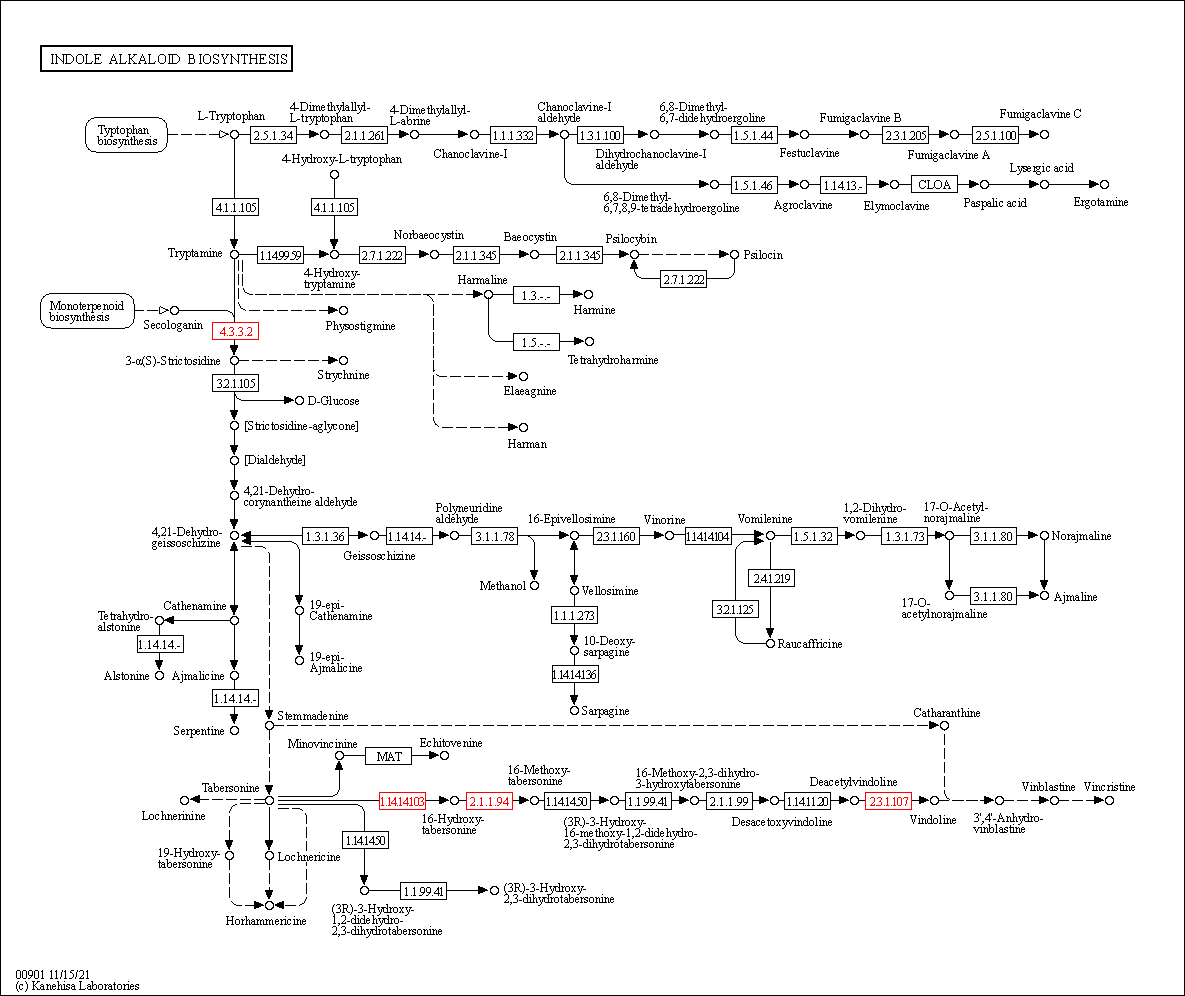


(B)
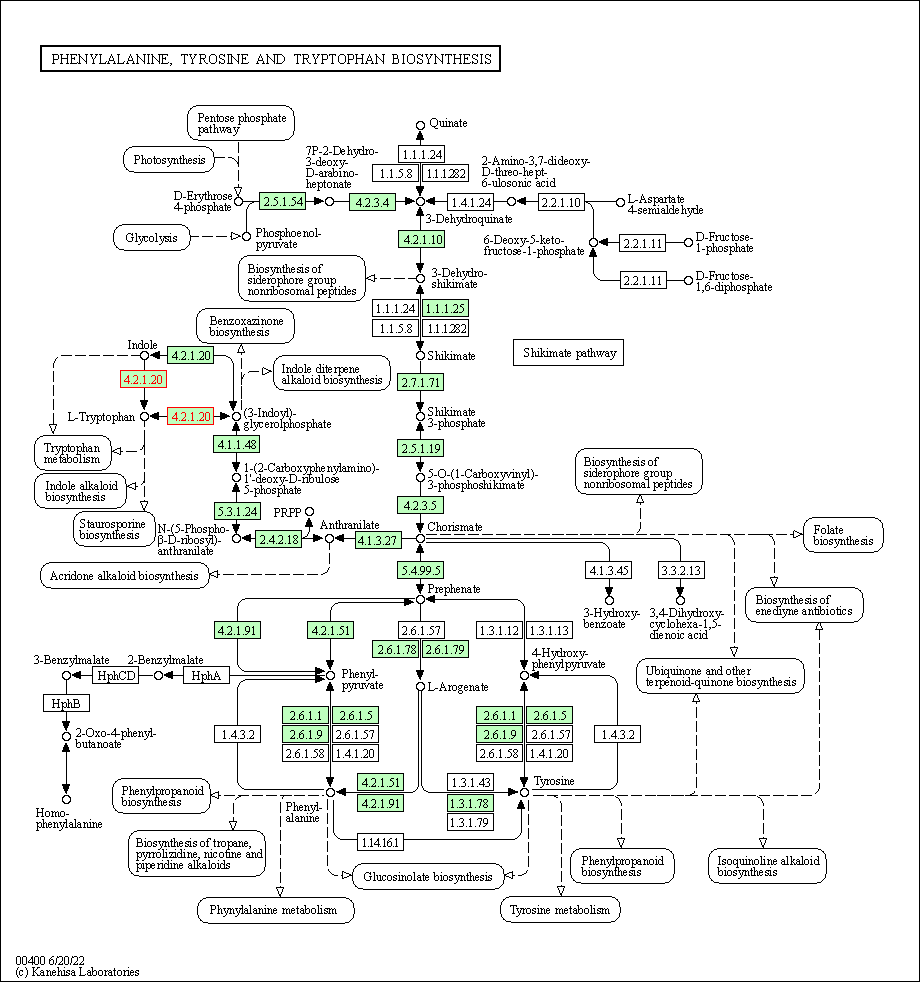


(C)


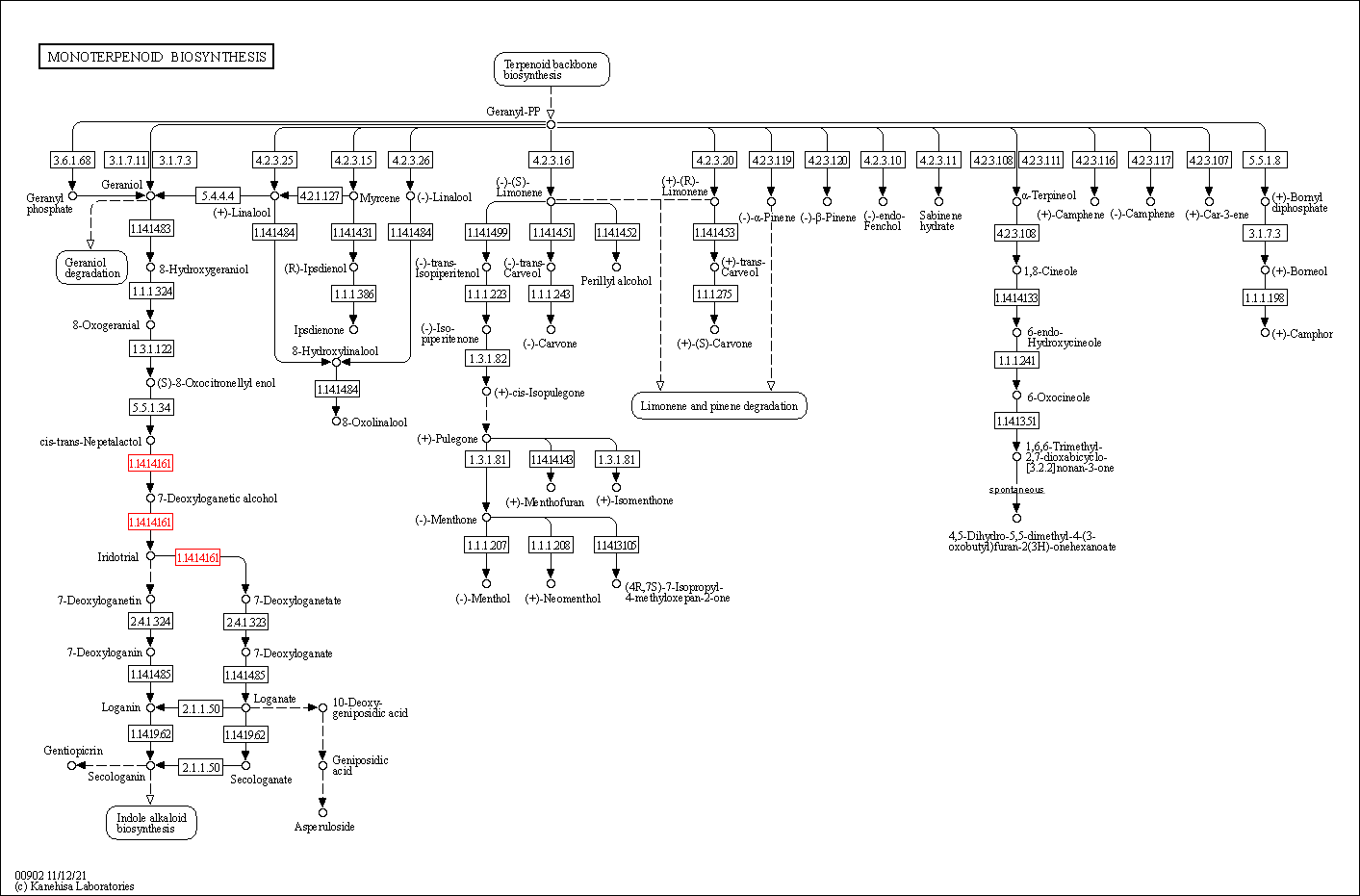


(D) *CRO_T107712* (ath00900)


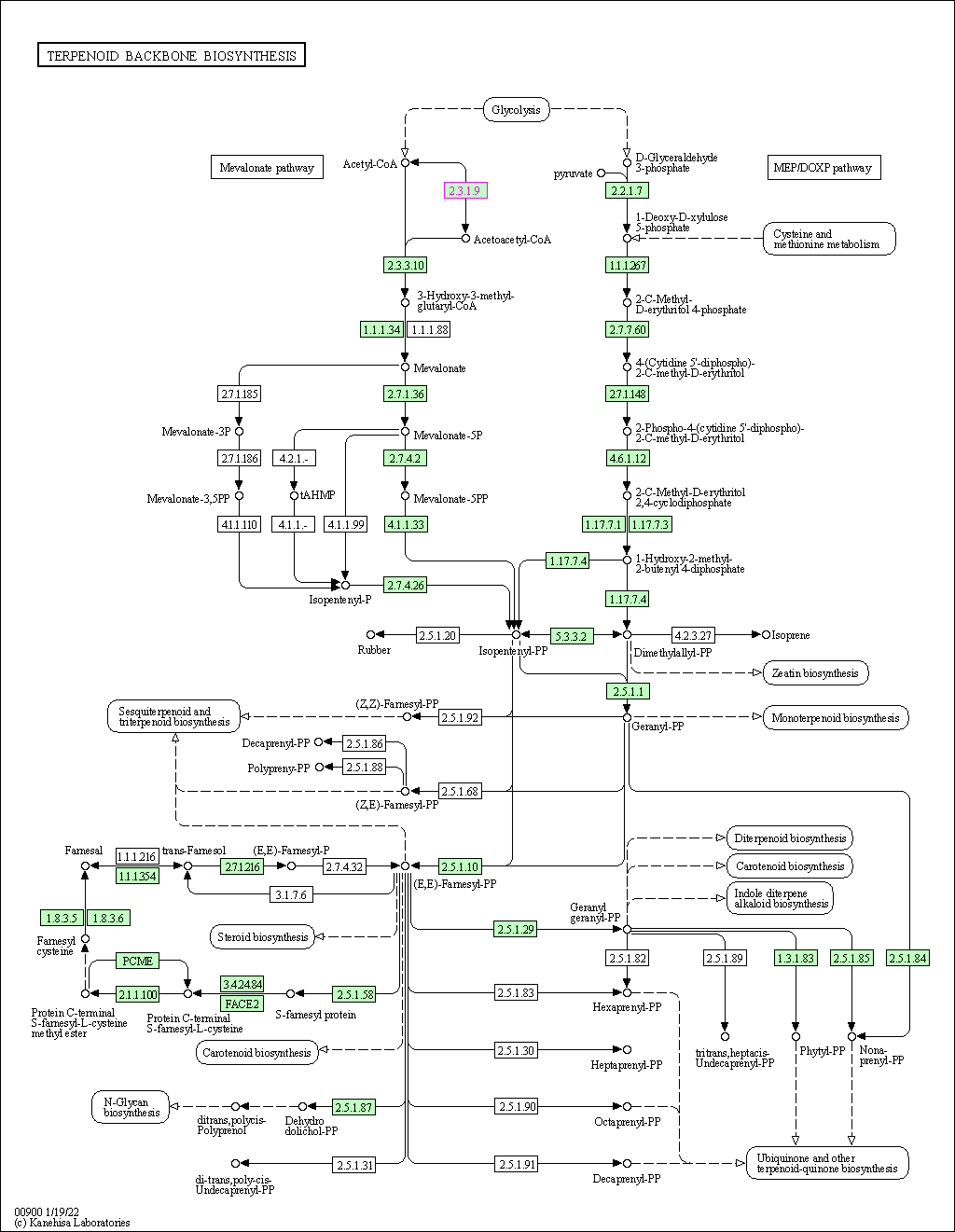


**Fig.  S3.** Enzymes coded by (A) *CRO_T120028* (map00901), (B) *CRO_T131457* (map00400), (C) *CRO_T138994* (map00902) and (D) *CRO_T107712* (ath00900) genes in the biosynthetic pathways related to TIAs production. Enzymes are shown by colored rectangles.
